# Endocrine responses to environmental variation

**DOI:** 10.1098/rstb.2022.0515

**Published:** 2024-03-25

**Authors:** Alexander G. Little, Frank Seebacher

**Affiliations:** ^1^ Department of Biology, Life Sciences Building, McMaster University, Hamilton, Ontario, Canada L8S 4K1; ^2^ School of Life and Environmental Sciences A08, University of Sydney, Sydney, New South Wales 2006, Australia

**Keywords:** thyroid, glucocorticoid, reproduction, endocrine flexibility, endocrine disrupting compounds, artificial light-at-night, climate change

## Abstract

Hormones regulate most physiological functions and life history from embryonic development to reproduction. In addition to their roles in growth and development, hormones also mediate responses to the abiotic, social and nutritional environments. Hormone signalling is responsive to environmental changes to adjust phenotypes to prevailing conditions. Both hormone levels and receptor densities can change to provide a flexible system of regulation. Endocrine flexibility connects the environment to organismal function, and it is central to understanding environmental impacts and their effect on individuals and populations. Hormones may also act as a ‘sensor’ to link environmental signals to epigenetic processes and thereby effect phenotypic plasticity within and across generations. Many environmental parameters are now changing in unprecedented ways as a result of human activity. The knowledge base of organism–environmental interactions was established in environments that differ in many ways from current conditions as a result of ongoing human impacts. It is an urgent contemporary challenge to understand how evolved endocrine responses will modulate phenotypes in response to anthropogenic environmental impacts including climate change, light-at-night and chemical pollution. Endocrine responses play a central role in ecology, and their integration into conservation can lead to more effective outcomes.

This article is part of the theme issue ‘Endocrine responses to environmental variation: conceptual approaches and recent developments’.

## 1. Introduction

**Figure 1.**
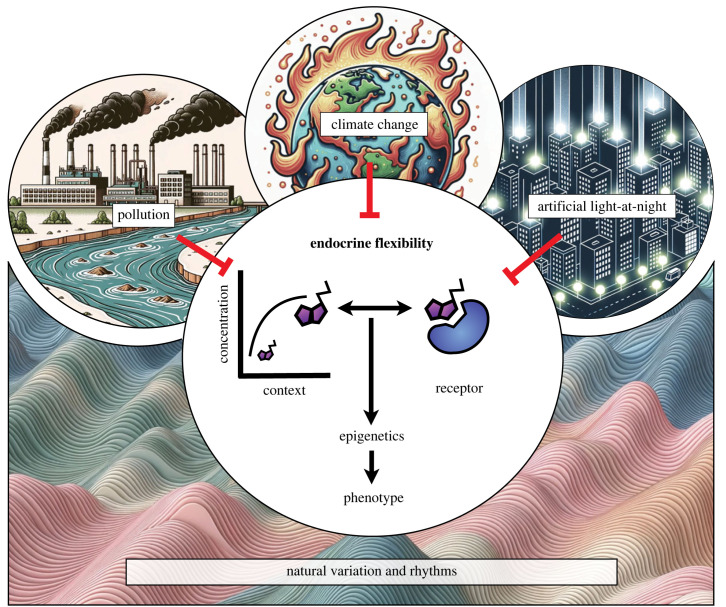
Schematic summary of the Theme Issue. Endocrine responses are dynamic and include context-dependent changes in the levels of circulating hormone and in hormone receptor densities. The resultant endocrine flexibility adjusts phenotypes directly or by inducing epigenetic modifications to match natural variation and rhythms. Anthropogenic impacts including pollution, artificial light-at-night and climate change can disrupt endocrine signalling to the detriment of organismal performance. (All images by the authors; some images were created with AI.)

Endocrine signalling regulates almost all biological processes, and the action of many hormones is closely linked to environmental signals. Hormones are therefore at the core of animal responses to environmental variation [1]. Endocrine systems are relevant ecologically through integrating environmental signals with fundamental biological responses that underpin individual fitness and the function of ecosystems [2–4]. Most hormones evolved to be multi-functional so that their effects on phenotypes can influence a broad range of ecological functions. Endocrine responses to environmental variability are inherently complex, requiring multidisciplinary research extending from molecular endocrinology to ecology and evolution [5]. For example, thyroid hormones regulate development and metamorphosis in amphibians and teleost fish, metabolic processes, and align physiological responses to seasonal environmental change [6]. Thyroid hormones may also act as environmental sensors to mediate plastic responses via DNA methylation [7]. Similarly, the hypothalamus–pituitary–adrenal (renal) axis and glucocorticoid signalling mediate metabolic responses to changes in the abiotic environment and can prepare an organism to respond to short-term biotic and abiotic stressors [8]. Glucocorticoids have featured prominently in research as biomarkers to assess acute and chronic stress responses [9]. A range of other hormones regulate various biological functions to match animal responses to environmental conditions. For example, leptin and gonadotropins regulate feeding and reproduction, respectively [10–12], and melatonin elicits a cascade of molecular responses to entrain a circadian sleep–wake cycle in response to light stimuli, which can even influence central thyroid processing ([13–15]; figure 1).

Anthropogenic endocrine disruption modifies endocrine-mediated responses to natural variation and links the fields of environmental science and toxicology to ecology and conservation biology [16,17]. Understanding of how anthropogenic impacts such as climate change, artificial light-at-night (ALAN) and endocrine disrupting chemicals (EDCs) alter evolved responses of animals is only now emerging [18]. There needs to be a collaborative effort to understand their impacts on non-human animals in the context of their natural and urbanized environments. The ultimate effects of natural and anthropogenic environmental variation (and disruption) on endocrine function are likely to determine the success of populations and species, so that understanding endocrine signalling and flexibility is important for effective conservation [19].

Here we briefly review endocrine responses to environmental variation, including measuring endocrine responses, endocrine flexibility and anthropogenic impacts on endocrine systems. Our mini-review serves to introduce the main themes of this Theme Issue.

## 2. How to measure endocrine responses?

There are different approaches to analysing hormone signalling, ranging from measures of circulating concentrations in the whole animal to analysing hormone receptor dynamics in local tissues [3,20]. Measuring circulating levels of hormones is relatively simple logistically, and samples can be taken from animals in the wild [21,22]. However, there is not always a clear correspondence between hormone concentrations and hormone signalling, because the latter may depend on receptor characteristics and densities rather than on circulating hormone levels alone [3,23]. Nuclear receptors such as thyroid and glucocorticoid receptors, in particular, are important in transducing endocrine signals by altering gene expression patterns [20,24,25]. It is essential therefore to develop frameworks to interpret hormone responses, and to establish the current state-of-knowledge of the link between various endocrine measures and animal function. For example, circulating glucocorticoid levels are commonly used to assess animal responses to challenging environments. However, glucocorticoid regulatory capacity depends on the interaction between circulating hormone and glucocorticoid receptor density [26]. The relationship between hormone levels and receptor densities can be predicted by a theoretical model tested against empirical data [26]. The model shows that individuals with higher glucocorticoid receptor densities had stronger physiological responses, and greater capacity to adjust physiological responses to environmental stressor intensity. Interestingly, variability in receptor density between individuals masked the correspondence between plasma glucocorticoid levels and physiological responses [26], indicating that plasma levels alone may be an insufficient indicator of stress responses. Ultimately, the crucial aspect of glucocorticoid signalling is its capacity to respond to and compensate for sudden changes in the environment [27], and this capacity is not necessarily reflected in isolated measures of circulating hormone levels. An alternative to measuring circulating hormone levels is the measurement of FKBP5, a protein in the glucocorticoid receptor complex that regulates receptor function and activity. FKBP5 sets the scope of glucocorticoid responses to environmental stressors [27]. Monitoring FKBP5 may therefore be a more effective way to assess endocrine flexibility and the efficacy of the physiological stress responses in individuals [27].

## 3. Endocrine flexibility

Global environments are inherently variable, and one of the principal challenges for most if not all organisms is to respond appropriately to environmental change, both with respect to the quality and the magnitude of the response. Environmental change represents a major selection pressure that can drive genetic adaptation. However, many environments are dynamic, and long-term mean changes are accompanied by shorter-term variation [28]. Hence, animals must have the capacity to respond relatively rapidly to external signals*.* Endocrine signals are the principal mechanisms that enable appropriate responses to dynamic environments, as encapsulated by the concept of endocrine flexibility [29–31]. Endocrine flexibility considers hormones as information carriers, which are deployed to effect within-individual rapid and reversible changes in physiological regulation in response to unpredictable challenges [4,32]. Useful information is contained in context-dependent changes in hormone concentrations coupled with receptor binding sensitivities, and individuals that can obtain the ‘best’ information about stressors will also be capable of mounting the most effective response [4,27].

Interestingly, hormone signalling may be intrinsically linked to epigenetic processes that can have lasting effects on phenotypes during the lifetime of an organism and across generations [33]. Epigenetic effects can have an endocrine dimension [34,35]. Hormone signals may enable epigenetic processes by acting as environmental sensors that transduce information about environmental change to epigenetic modifications such as DNA methylation patterns. Thyroid response elements in the promotor region of the genes coding for the principal enzymes underlying DNA methylation, for example, can link sensing of light and temperature by thyroid hormone to DNA methylation patterns [7]. Hence, endocrine flexibility may comprise information transfer by hormones leading to epigenetic changes of phenotypes in response to environmental variability.

## 4. Endocrine responses to anthropogenic impacts

The three-way interaction between environmental change, hormones and epigenetic processes can have complex impacts on animal function. Given the complexity of evolved endocrine responses, disruption of hormone signalling is likely to produce unexpected and unpredictable effects [36,37]. Most ecosystems in the world are experiencing unprecedented environmental change as a result of human activity. In addition to climate change, ALAN and endocrine disrupting chemicals (EDCs) are two of the most dominant anthropogenic impacts. All three are currently increasing at an alarming rate [38,39], and all three can impact hormone-mediated signalling [40–42]. Importantly, these anthropogenic drivers can interact and alter qualitatively how animals respond to natural variation: existing knowledge of responses to natural environmental variability may therefore be outdated [37,43,44]. Hence, there is considerable urgency to assess how animal responses are altered in the presence of endocrine disruption to facilitate effective conservation [45].

ALAN disrupts biological clocks, which are evolved time-keeping systems that align physiology with daily or seasonal rhythms in environmental temperature and light [15]. Biological clocks are regulated by melatonin and thyroid hormone, and physiological responses are coordinated via glucocorticoid levels and receptors [15,46–48]. ALAN is increasing globally as a result of increasing urbanisation and human activity [49,50] and its disruption of the endocrine-mediated time-keeping system can have pronounced effects on reproduction, growth and behaviour, and thereby reduce fitness and ecosystem function [51–53]

Similarly, environmental concentrations of EDCs, such as bisphenols derived from plastic waste and manufacture, are increasing at an alarming rate worldwide [18,54,55]. EDCs interact with nuclear receptors such as thyroid and glucocorticoid receptors to activate or block endocrine signals [25,56,57]. EDCs thereby impair essential biological functions across a broad range of taxa [58] and have the potential to disrupt ecosystems, particularly in aquatic environments [18]. Environmental exposures throughout the lifetime define the chemical exposome of an organism [59]. The concept originates from medicine to evaluate environmental impacts on human health but has been extended to the eco-exposome that includes non-human systems [60]. The exposome, or components of it, can impact endocrine systems and thereby phenotypes [61]. Organisms have evolved metabolic pathways to eliminate xenobiotics, but detoxification is relatively non-specific and can cause side effects including break-down of endogenous hormones [16].

Climate change and its associated increases in average temperatures and temperature fluctuations can impact animals directly by temperature-induced impairment of physiological rate functions, and of reproductive function and fitness [62]. Endocrine flexibility has the potential to alleviate the negative effects of warming and increased variability, in particular via glucocorticoid signalling [17]. However, there are likely to be taxonomic differences and limits in its efficacy to respond to extreme environments such as urban heat islands. Future research should be directed to increase understanding of the endocrine mechanisms that allow animals to cope with variable conditions, which will help identify the populations that are most vulnerable to climate change [17]. Interestingly, an analysis of glucocorticoid concentrations in 51 species of birds in the USA showed a landscape-level association between stress-induced levels of glucocorticoids and usable land cover within and across species [63]. These data indicate that glucocorticoid levels may be a useful biomarker to characterize populations across their range of available habitats, which can feed into informing conservation strategies. Climate change and increasing temperatures can also interact with endocrine signalling to alter body size and shape [64]. Additionally, warming can exacerbate the effects of EDCs [43] and intensify their negative impact on size, growth and metabolism [65]. These compounded effects could disrupt energy transfer between trophic levels and should be incorporated into population models such as fisheries models [66].

Endocrine signalling is an essential mechanism by which organisms respond to environmental change and potentially alleviate its negative effects. Endocrine responses are therefore an essential component in evolution, ecology and conservation. In the face of rapidly changing global environments, conservation efforts can only achieve a fraction of their effectiveness if endocrine responses are not incorporated explicitly.

## Data Availability

This article has no additional data.

## References

[1] Wingfield JC. 2008 Comparative endocrinology, environment and global change. Gen. Comp. Endocr. **157**, 207-216. (10.1016/j.ygcen.2008.04.017) 18558405

[2] Lema SC. 2020 Editorial: The adaptive value of hormones: endocrine systems as outcomes and initiators of evolution. Mol. Cell. Endocrinol. **517**, 110983. (10.1016/j.mce.2020.110983) 32781249

[3] Schoenle LA, Zimmer C, Miller ET, Vitousek MN. 2021 Does variation in glucocorticoid concentrations predict fitness? A phylogenetic meta-analysis. Gen. Comp. Endocr. **300**, 113611. (10.1016/j.ygcen.2020.113611) 32950580

[4] Zimmer C, Woods HA, Martin LB. 2022 Information theory in vertebrate stress physiology. Trends Endocrinol. Metab. **33**, 8-17. (10.1016/j.tem.2021.10.001) 34750063

[5] Orr JA et al. 2020 Towards a unified study of multiple stressors: divisions and common goals across research disciplines. Proc. R. Soc. B **287**, 20200421. (10.1098/rspb.2020.0421) PMC7282922 32370677

[6] Zwahlen J, Gairin E, Vianello S, Mercader M, Roux N, Laudet V. 2024 The ecological function of thyroid hormones. Phil. Trans. R. Soc. B **379**, 20220511. (10.1098/rstb.2022.0511)38310932

[7] Seebacher F, Little AG. 2024 Thyroid hormone links environmental signals to DNA methylation. Phil. Trans. R. Soc. B **379**, 20220506. (10.1098/rstb.2022.0506)38310936

[8] Bruijn R de, Romero LM. 2018 The role of glucocorticoids in the vertebrate response to weather. Gen. Comp. Endocrinol. **269**, 11-32. (10.1016/j.ygcen.2018.07.007) 30012539

[9] Injaian AS et al. 2020 Baseline and stress-induced corticosterone levels across birds and reptiles do not reflect urbanization levels. Conserv. Physiol. **8**, coz110. (10.1093/conphys/coz110) 31993201 PMC6978728

[10] Brüning A, Hölker F, Franke S, Kleiner W, Kloas W. 2016 Impact of different colours of artificial light at night on melatonin rhythm and gene expression of gonadotropins in European perch. Sci. Total Environ. **543**, 214-222. (10.1016/j.scitotenv.2015.11.023) 26584071

[11] Volkoff H. 2019 Fish as models for understanding the vertebrate endocrine regulation of feeding and weight. Mol. Cell. Endocrinol. **497**, 110437-13. (10.1016/j.mce.2019.04.017) 31054868

[12] Volkoff H. 2024 The effects of environmental changes on the endocrine regulation of feeding in fishes. Phil. Trans. R. Soc. B **379**, 20220503. (10.1098/rstb.2022.0503)38310931

[13] Dardente H, Wyse CA, Birnie MJ, Dupré SM, Loudon ASI, Lincoln GA, Hazlerigg DG. 2010 A molecular switch for photoperiod responsiveness in mammals. Curr. Biol. **20**, 2193-2198. (10.1016/j.cub.2010.10.048) 21129971

[14] Huffeldt NP, Merkel FR, Jenni-Eiermann S, Goymann W, Helm B. 2020 Melatonin and corticosterone profiles under polar day in a seabird with sexually opposite activity-rhythms. Gen. Comp. Endocr. **285**, 113296. (10.1016/j.ygcen.2019.113296) 31589833

[15] Helm B, Greives T, Zeman M. 2024 Endocrine–circadian interactions in birds: implications when nights are no longer dark. Phil. Trans. R. Soc. B **379**, 20220514. (10.1098/rstb.2022.0514)38310930

[16] Tomkiewicz C, Coumoul X, Nioche P, Barouki R, Blanc EB. 2024 Costs of molecular adaptation to the chemical exposome: a focus on xenobiotic metabolism pathways. Phil. Trans. R. Soc. B **379**, 20220510. (10.1098/rstb.2022.0510)38310928

[17] Taff CC, Baldan D, Mentesana L, Ouyang JQ, Vitousek MN, Hau M. 2024 Endocrine flexibility can facilitate or constrain the ability to cope with global change. Phil. Trans. R. Soc. B **379**, 20220502. (10.1098/rstb.2022.0502) PMC10838644 38310929

[18] Kloas W, Stöck M, Lutz I, Ziková-Kloas A. 2024 Endocrine disruption in teleosts and amphibians is mediated by anthropogenic and natural environmental factors: implications for risk assessment. Phil. Trans. R. Soc. B **379**, 20220505. (10.1098/rstb.2022.0505)38310939

[19] Hölker F et al. 2021 11 Pressing research questions on how light pollution affects biodiversity. Front. Ecol. Evol. **9**, 767177. (10.3389/fevo.2021.767177)

[20] Holzer G, Roux N, Laudet V. 2017 Evolution of ligands, receptors and metabolizing enzymes of thyroid signaling. Mol. Cell. Endocrinol. **459**, 5-13. (10.1016/j.mce.2017.03.021) 28342854

[21] Dominoni DM, Teo D, Branston CJ, Jakhar A, Albalawi BFA, Evans NP. 2021 Feather, but not plasma, glucocorticoid response to artificial light at night differs between urban and forest blue tit nestlings. Integr. Comp. Biol. **61**, 1111-1112. (10.1093/icb/icab067) 34272860 PMC8490687

[22] Narayan EJ, Forsburg ZR, Davis DR, Gabor CR. 2019 Non-invasive methods for measuring and monitoring stress physiology in imperiled amphibians. Front. Ecol. Evol. **7**, 431. (10.3389/fevo.2019.00431)

[23] Romero LM, Beattie UK. 2022 Common myths of glucocorticoid function in ecology and conservation. J. Exp. Zool. A **337**, 7-14. (10.1002/jez.2459) 33819389

[24] Little AG. 2018 Local regulation of thyroid hormone signaling. Vitam. Horm. **106**, 1-17. (10.1016/bs.vh.2017.06.004) 29407431

[25] Miglioli A, Fonseca E, Besnardeau L, Canesi L, Schubert M, Dumollard R. 2024 First characterization of the nuclear receptor superfamily in the Mediterranean mussel *Mytilus galloprovincialis*: developmental expression dynamics and potential susceptibility to environmental chemicals. Phil. Trans. R. Soc. B **379**, 20220500. (10.1098/rstb.2022.0500)38310933

[26] Jimeno B, Rubalcaba JG. 2024 Modelling the role of glucocorticoid receptor as mediator of endocrine responses to environmental challenge. Phil. Trans. R. Soc. B **379**, 20220501. (10.1098/rstb.2022.0501)38310935

[27] Zimmer C, Jimeno B, Martin LB. 2024 HPA flexibility and *FKBP5*: promising physiological targets for conservation. Phil. Trans. R. Soc. B **379**, 20220512. (10.1098/rstb.2022.0512)38310934

[28] Hofmeister NR, Rubenstein DR. 2016 Environmental variability and the evolution of the glucocorticoid receptor (Nr3c1) in African starlings. Ecol. Lett. **19**, 1219-1227. (10.1111/ele.12656) 27500971

[29] Taff CC, Vitousek MN. 2016 Endocrine flexibility: optimizing phenotypes in a dynamic world? Trends Ecol. Evol. **31**, 476-488. (10.1016/j.tree.2016.03.005) 27055729

[30] Grindstaff JL, Beaty LE, Ambardar M, Luttbeg B. 2022 Integrating theoretical and empirical approaches for a robust understanding of endocrine flexibility. J. Exp. Biol. **225**, jeb243408. (10.1242/jeb.243408) 35258612 PMC8987727

[31] Lema SC. 2020 Hormones, developmental plasticity, and adaptive evolution: endocrine flexibility as a catalyst for ‘plasticity-first’ phenotypic divergence. Mol. Cell. Endocrinol. **502**, 110678. (10.1016/j.mce.2019.110678) 31830511

[32] Martin LB, Zimmer C. 2022 Endocrine flexibility. J. Exp. Biol. **225**, jeb.244646. (10.1242/jeb.244646) 36017760

[33] Radford EJ. 2018 Exploring the extent and scope of epigenetic inheritance. Nat. Rev. Endocrinol. **14**, 345-355. (10.1038/s41574-018-0005-5) 29666451

[34] Zhu L, Liu Y, Xue X, Yuan C, Wang Z. 2021 BPA's transgenerational disturbance to transcription of ovarian steroidogenic genes in rare minnow *Gobiocypris rarus* via DNA and histone methylation. Sci. Total Environ. **762**, 143055. (10.1016/j.scitotenv.2020.143055) 33127149

[35] Raj S, Kyono Y, Sifuentes CJ, Arellanes-Licea E del C, Subramani A, Denver RJ. 2020 Thyroid hormone induces dna demethylation in *Xenopus* tadpole brain. Endocrinology **161**, bqaa155. (10.1210/endocr/bqaa155) 32865566 PMC7947600

[36] Dominoni D, Smit JAH, Visser ME, Halfwerk W. 2020 Multisensory pollution: Artificial light at night and anthropogenic noise have interactive effects on activity patterns of great tits (*Parus major*). Environ. Pollut. **256**, 113314. (10.1016/j.envpol.2019.113314) 31761596

[37] Polazzo F, Roth SK, Hermann M, Mangold-Döring A, Rico A, Sobek A, Brink PJV den, Jackson MC. 2022 Combined effects of heatwaves and micropollutants on freshwater ecosystems: Towards an integrated assessment of extreme events in multiple stressors research. Glob. Change Biol. **28**, 1248-1267. (10.1111/gcb.15971) PMC9298819 34735747

[38] Borrelle SB et al. 2020 Predicted growth in plastic waste exceeds efforts to mitigate plastic pollution. Science **369**, 1515-1518. (10.1126/science.aba3656) 32943526

[39] Sanders D, Frago E, Kehoe R, Patterson C, Gaston KJ. 2020 A meta-analysis of biological impacts of artificial light at night. Nat. Ecol. Evol. **5**, 74-81. (10.1038/s41559-020-01322-x) 33139919

[40] Hooper MJ, Ankley GT, Cristol DA, Maryoung LA, Noyes PD, Pinkerton KE. 2013 Interactions between chemical and climate stressors: A role for mechanistic toxicology in assessing climate change risks. Environ. Toxicol. Chem. **32**, 32-48. (10.1002/etc.2043) 23136056 PMC3601417

[41] Besson M et al. 2020 Anthropogenic stressors impact fish sensory development and survival via thyroid disruption. Nat. Commun. **11**, 3614. (10.1038/s41467-020-17450-8) 32681015 PMC7367887

[42] Russart KLG, Nelson RJ. 2018 Light at night as an environmental endocrine disruptor. Physiol. Behav. **190**, 82-89. (10.1016/j.physbeh.2017.08.029) 28870443 PMC5839924

[43] Little AG, Seebacher F. 2015 Temperature determines toxicity: Bisphenol A reduces thermal tolerance in fish. Environ. Poll. **197**, 84-89. (10.1016/j.envpol.2014.12.003) 25514059

[44] Seebacher F. 2022 Interactive effects of anthropogenic environmental drivers on endocrine responses in wildlife. Mol. Cell. Endocrinol. **556**, 111737. (10.1016/j.mce.2022.111737) 35931299

[45] Sutherland WJ et al. 2021 A 2021 Horizon scan of emerging global biological conservation issues. Trends Ecol. Evol. **36**, 87-97. (10.1016/j.tree.2020.10.014) 33213887

[46] Jaikumar G, Slabbekoorn H, Sireeni J, Schaaf M, Tudorache C. 2020 The role of the glucocorticoid receptor in the regulation of diel rhythmicity. Physiol. Behav. **223**, 112991-8. (10.1016/j.physbeh.2020.112991) 32497529

[47] Grubisic M et al. 2019 Light pollution, circadian photoreception, and melatonin in vertebrates. Sustainability **11**, 6400. (10.3390/su11226400)

[48] Kupprat F, Kloas W, Krüger A, Schmalsch C, Hölker F. 2021 Misbalance of thyroid hormones after two weeks of exposure to artificial light at night in Eurasian perch *Perca fluviatilis*. Conserv. Physiol. **9**, coaa124. (10.1093/conphys/coaa124) 33659060 PMC7905158

[49] Falchi F, Cinzano P, Duriscoe D, Kyba CCM, Elvidge CD, Baugh K, Portnov BA, Rybnikova NA, Furgoni R. 2016 The new world atlas of artificial night sky brightness. Sci. Adv. **2**, e1600377-27. (10.1126/sciadv.1600377) 27386582 PMC4928945

[50] Falcón J, Torriglia A, Attia D, Viénot F, Gronfier C, Behar-Cohen F, Martinsons C, Hicks D. 2020 Exposure to artificial light at night and the consequences for flora, fauna, and ecosystems. Front. Neurosci. **14**, 602796. (10.3389/fnins.2020.602796) 33304237 PMC7701298

[51] Jägerbrand AK, Spoelstra K. 2023 Effects of anthropogenic light on species and ecosystems. Science **380**, 1125-1130. (10.1126/science.adg3173) 37319223

[52] Miner KA, Huertas M, Aspbury AS, Gabor CR. 2021 Artificial light at night alters the physiology and behavior of western mosquitofish (*Gambusia affinis*). Front. Ecol. Evol. **9**, 617063. (10.3389/fevo.2021.617063)

[53] Willems JS, Phillips JN, Francis CD. 2022 Artificial light at night and anthropogenic noise alter the foraging activity and structure of vertebrate communities. Sci. Total Environ. **805**, 150223. (10.1016/j.scitotenv.2021.150223) 34537710

[54] Subramanian M. 2022 Can nations reign in plastic pollution? Nature **611**, 650-653. (10.1038/d41586-022-03793-3) 36414780

[55] Tarafdar A et al. 2022 The hazardous threat of Bisphenol A: Toxicity, detection and remediation. J. Hazard. Mater. **423**, 127097. (10.1016/j.jhazmat.2021.127097) 34488101

[56] Lu L, Zhan T, Ma M, Xu C, Wang J, Zhang C, Liu W, Zhuang S. 2018 Thyroid disruption by bisphenol s analogues via thyroid hormone receptor *β*: *In vitro*, *in vivo*, and molecular dynamics simulation study. Environ. Sci. Technol. **52**, 6617-6625. (10.1021/acs.est.8b00776) 29763311

[57] Beg MA, Sheikh IA. 2020 Endocrine disruption: Molecular interactions of environmental bisphenol contaminants with thyroid hormone receptor and thyroxine-binding globulin. Toxicol. Ind. Health **36**, 322-335. (10.1177/0748233720928165) 32496146

[58] Wu NC, Seebacher F. 2020 Effect of the plastic pollutant bisphenol A on the biology of aquatic organisms: A meta-analysis. Glob. Change Biol. **26**, 3821-3833. (10.1111/gcb.15127) 32436328

[59] Barouki R et al. 2021 The exposome and toxicology: A win–win collaboration. Toxicol. Sci. **186**, 1-11. (10.1093/toxsci/kfab149) PMC9019839 34878125

[60] Scholz S et al. 2022 The eco-exposome concept: Supporting an integrated assessment of mixtures of environmental chemicals. Environ. Toxicol. Chem. **41**, 30-45. (10.1002/etc.5242) 34714945 PMC9104394

[61] Barouki R. 2010 Linking long-term toxicity of xeno-chemicals with short-term biological adaptation. Biochimie **92**, 1222-1226. (10.1016/j.biochi.2010.02.026) 20188785

[62] Lema SC, Luckenbach JA, Yamamoto Y, Housh MJ. 2024 Fish reproduction in a warming world: vulnerable points in hormone regulation from sex determination to spawning. Phil. Trans. R. Soc. B **379**, 20220516. (10.1098/rstb.2022.0516)38310938

[63] Alaasam VJ, Behnke TL, Grant AR, Ouyang JQ. 2024 Glucocorticoids and land cover: a largescale comparative approach to assess a physiological biomarker for avian conservation. Phil. Trans. R. Soc. B **379**, 20220508. (10.1098/rstb.2022.0508)38310940

[64] Names GR, Grindstaff JL, Westneat DF, Heidinger BJ. 2024 Climate change and its effects on body size and shape: the role of endocrine mechanisms. Phil. Trans. R. Soc. B **379**, 20220509. (10.1098/rstb.2022.0509)38310941

[65] Wu NC, Rubin AM, Seebacher F. 2022 Endocrine disruption from plastic pollution and warming interact to increase the energetic cost of growth in a fish. Proc. R. Soc. B **289**, 20212077. (10.1098/rspb.2021.2077) PMC8790379 35078359

[66] Gaines SD et al*.* 2018 Improved fisheries management could offset many negative effects of climate change. Sci. Adv. **4**, eaao1378. (10.1126/sciadv.aao1378) 30167455 PMC6114984

